# Supplementary material for: Mutation of OsLPR3 Enhances Tolerance to Phosphate Starvation in Rice
Source: Int J Mol Sci. 2023 Jan 26;24(3):2437. doi: 10.3390/ijms24032437 (PMC9917114; doi:10.3390/ijms24032437)
Supplement: Supplementary file 1 [file ijms-24-02437-s001.zip › ijms-2140334-supplementary.pdf]

Supplementary Figures

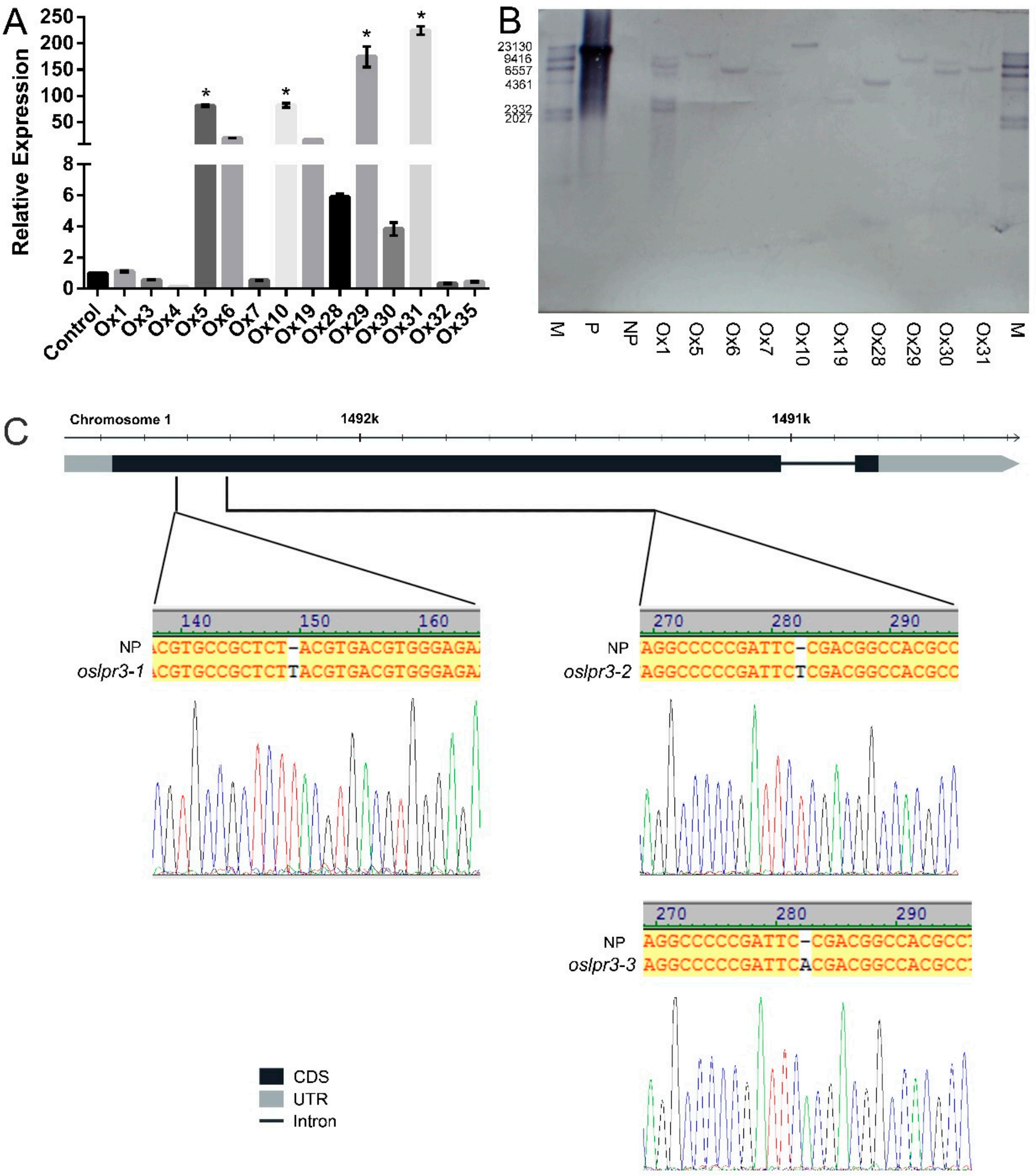

Fig. S1. Identification of *OsLPR3* transgenic lines.

WT and OsLPR3 transgenic lines were grown hydroponically in +P medium for 2 weeks. (A) qRT-PCR assays were performed to determine the relative expression levels of *OsLPR3* transcripts in the roots. *OsActin* was used as an internal control. Values are means ± SE (n = 3). Different letters above the bars indicate significant differences ($P < 0.05$, one-way ANOVA). (B) Southern blot analysis of *OsLPR3* overexpression lines. (C) Total DNA of *oslpr3* mutants were isolated from leaf blade tissues and identified by sequencing. The *OsLPR3* gene structure is shown at the top. The coding sequence (CDS), untranslated regions (UTRs), and introns are indicated by black boxes, gray boxes, and a black bar, respectively. The mutation site of three independent knockout lines and a comparison of their nucleotide sequence to that of WT are shown. Below each sequence is a sequencing chromatograph for the *OsLPR3* mutant.

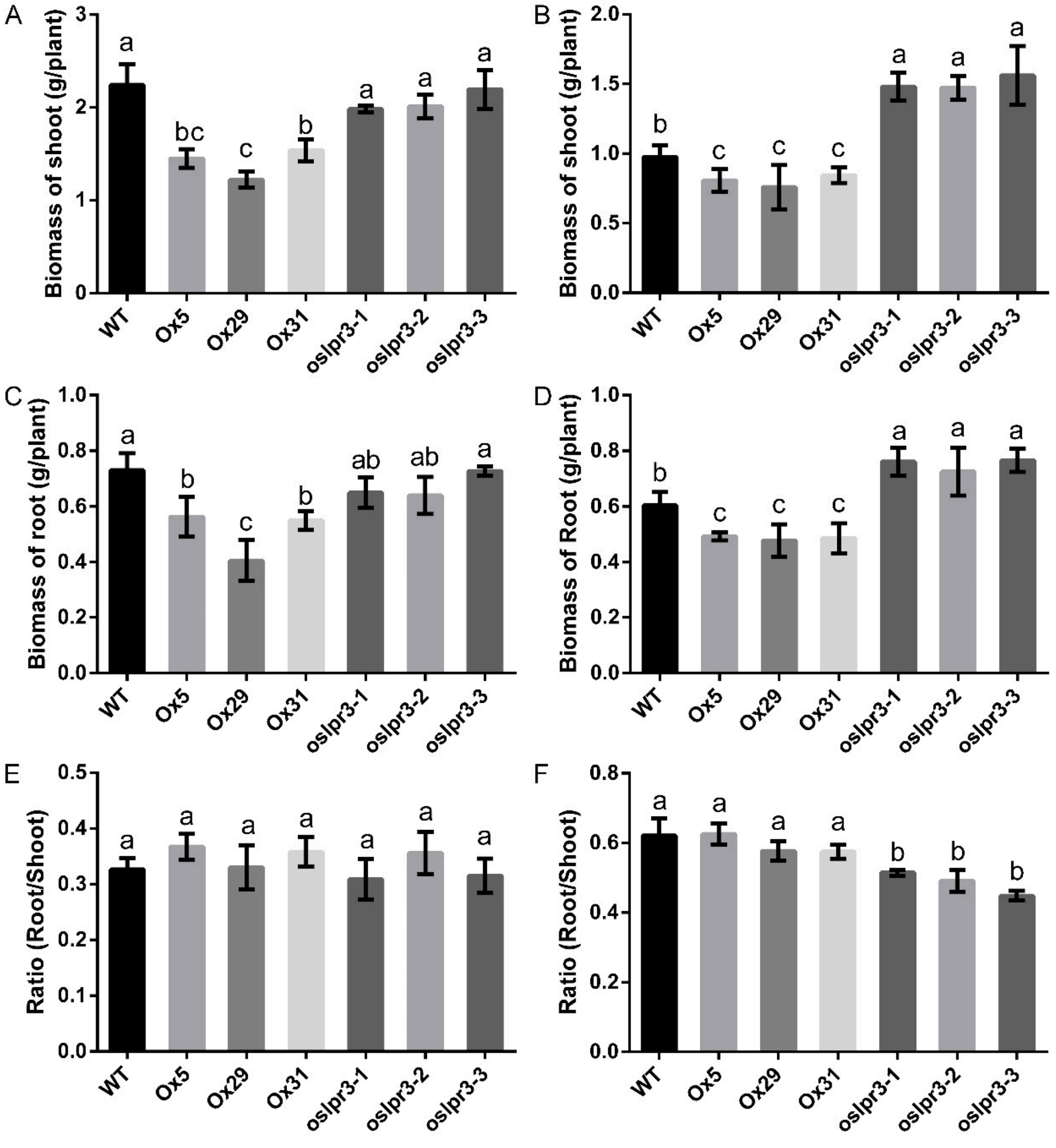

Fig. S2. Biomass and root–shoot ratio of WT, *OsLPR3*-Ox, and *oslpr3* knockout lines at different Pi supplies.

Seedlings were grown hydroponically as described in Fig. 3. Shoot biomass, root biomass, and root–shoot ratio under +P conditions (A, B, and C) and under −P conditions (D, E, and F). Values are means ± SE (n = 6). Different letters above the bars indicate significant differences (P < 0.05, one-way ANOVA).

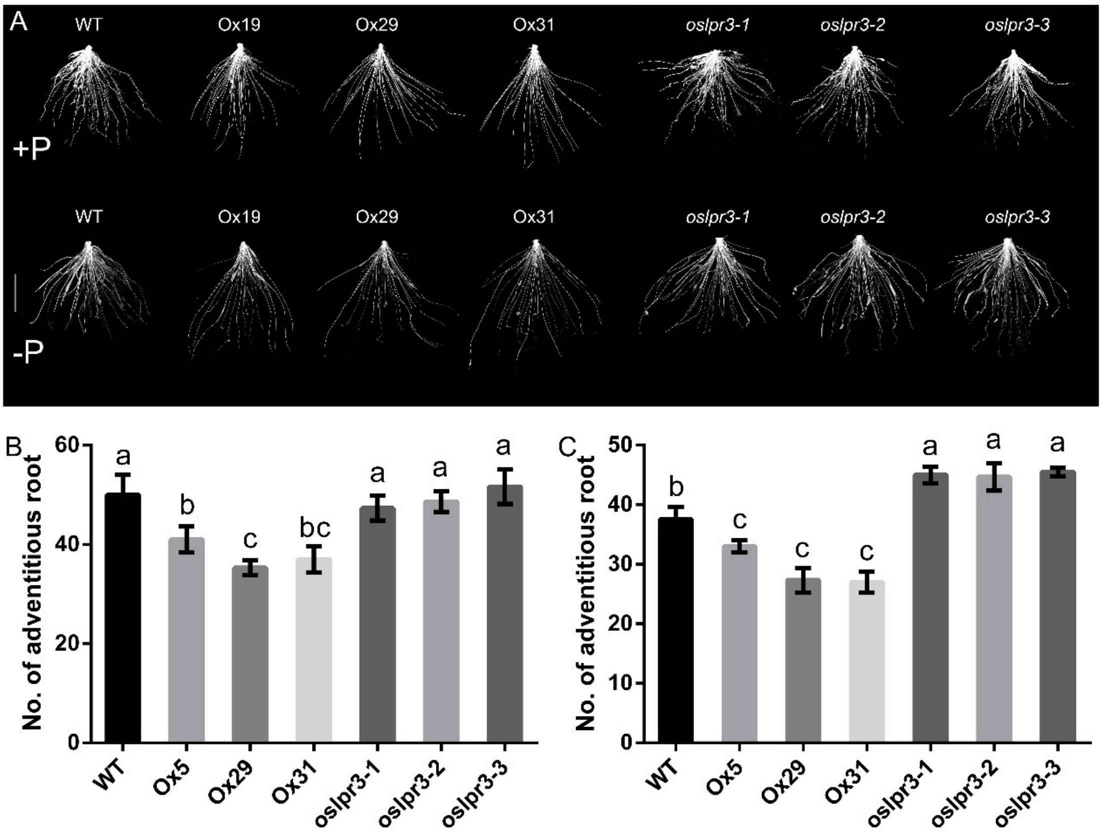

Fig. S3. Altered *OsLPR3* expression affects the number of adventitious roots.

Seedlings (10-d-old) of WT, *OsLPR3*-Ox, and *oslpr3* lines were grown hydroponically under +P and −P conditions for 21 d. (A) Root scans showing the root phenotypes of WT, OsLPR3-Ox, and *oslpr3* plants grown under +P conditions (top row) and −P conditions (bottom row). Scale bar = 5 cm. (B and C) Number of adventitious roots in WT, OsLPR3-Ox, and *oslpr3* lines under +P and −P conditions, respectively. Values are means ± SE (n = 6). Different letters above the bars indicate significant differences ($P < 0.05$, one-way ANOVA).

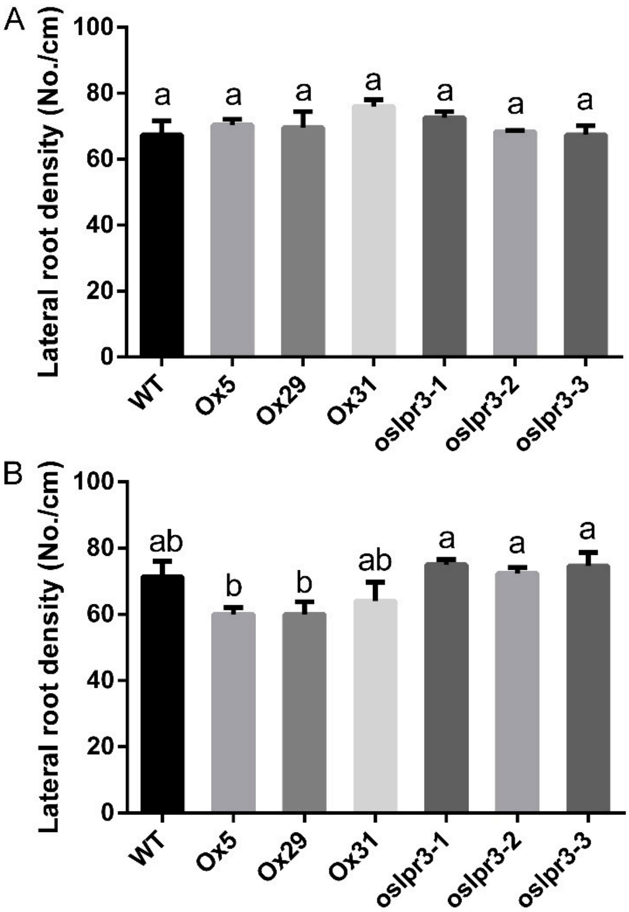

Fig. S4. Lateral root density of WT, *OsLPR3*-Ox, and *oslpr3* knockout lines at different Pi supplies. Seedlings were grown hydroponically as described in Fig.3. Lateral root density was measured under +P conditions (A) and −P conditions (B) using ImageJ. Values are means ± SE (n = 6). Different letters above the bars indicate significant differences (P < 0.05, one-way ANOVA).

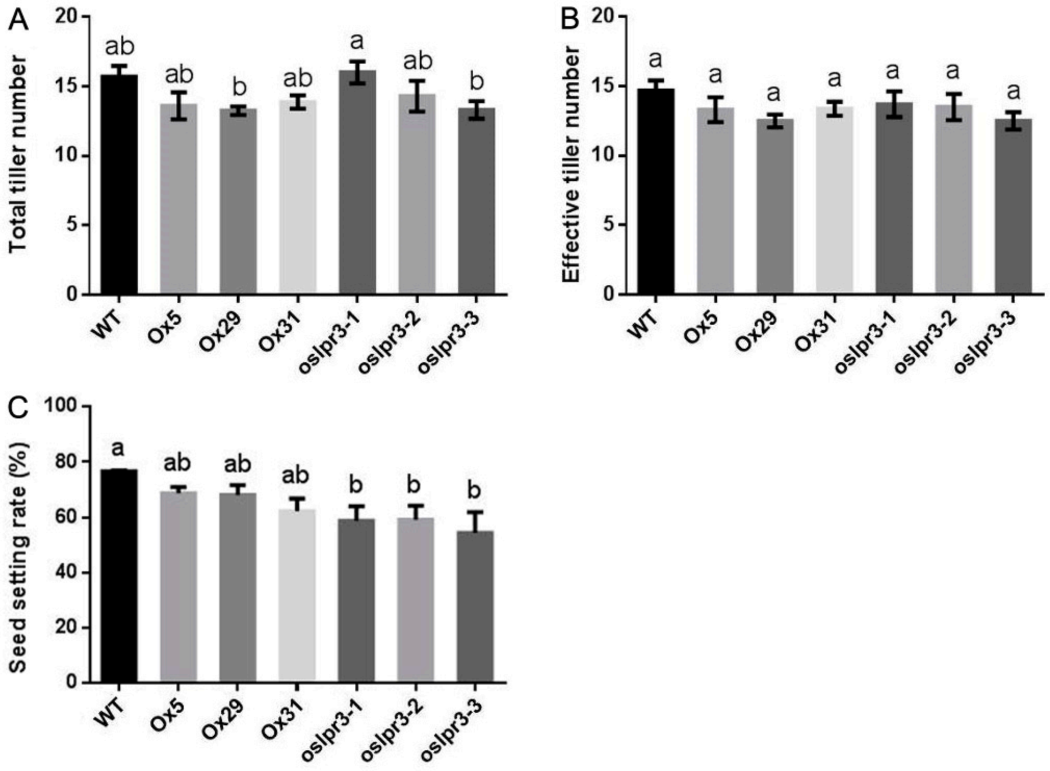

Fig. S5. The **affects of *OsLPR3* on tiller number and seed setting rate.**

WT, *OsLPR3*-Ox, and *oslpr3* lines were grown to maturity (20 weeks), total tiller number (A), effective tiller number (B) and seed setting rate (B) were determined. Values are means ± SE (*n* = 10). Different letters above the bars indicate significant differences (*P* < 0.05, one-way ANOVA).

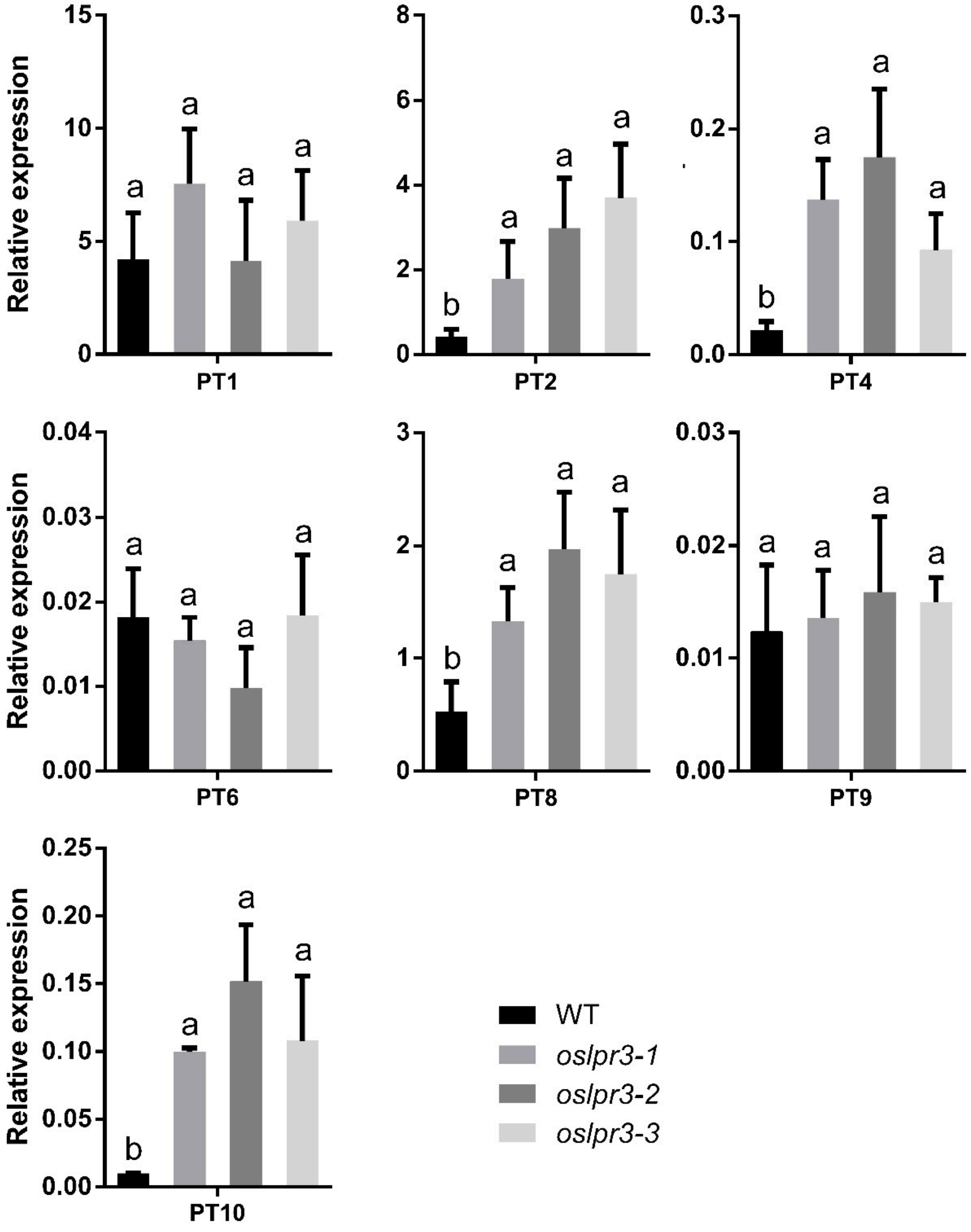

Fig. S6. Expression of the seven members of the rice PHT family in wild-type (WT) and *oslpr3* mutant plants under +P conditions.

Seedlings (10-d-old) of WT and *oslpr3* lines were grown hydroponically under +P conditions for 10 d. Relative OsPT transcript levels in the roots were determined and compared with *OsActin* by qPCR. Values are means ± SE (n = 3). Different letters above the bars indicate significant differences ($P < 0.05$, one-way ANOVA).

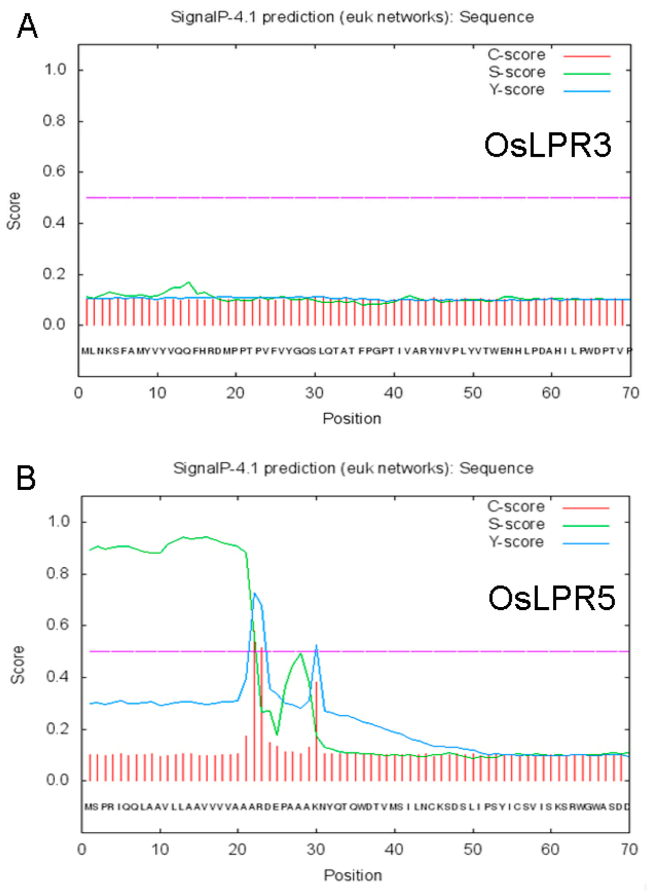

Fig. S7. Signal peptide analysis of OsLPR3 and OsLPR5.

Amino acid sequences of OsLPR3 (A) and OsLPR5 (B) were submitted to SignalP 4.1 (www.cbs.dtu.dk/services/SignalP/). C-score was used to distinguish whether it was a clipping site, S-score was used to determine whether the corresponding location was a signal peptide region, and Y-score was the geometric mean of C-score and S-score, representing the combined cleavage site score.

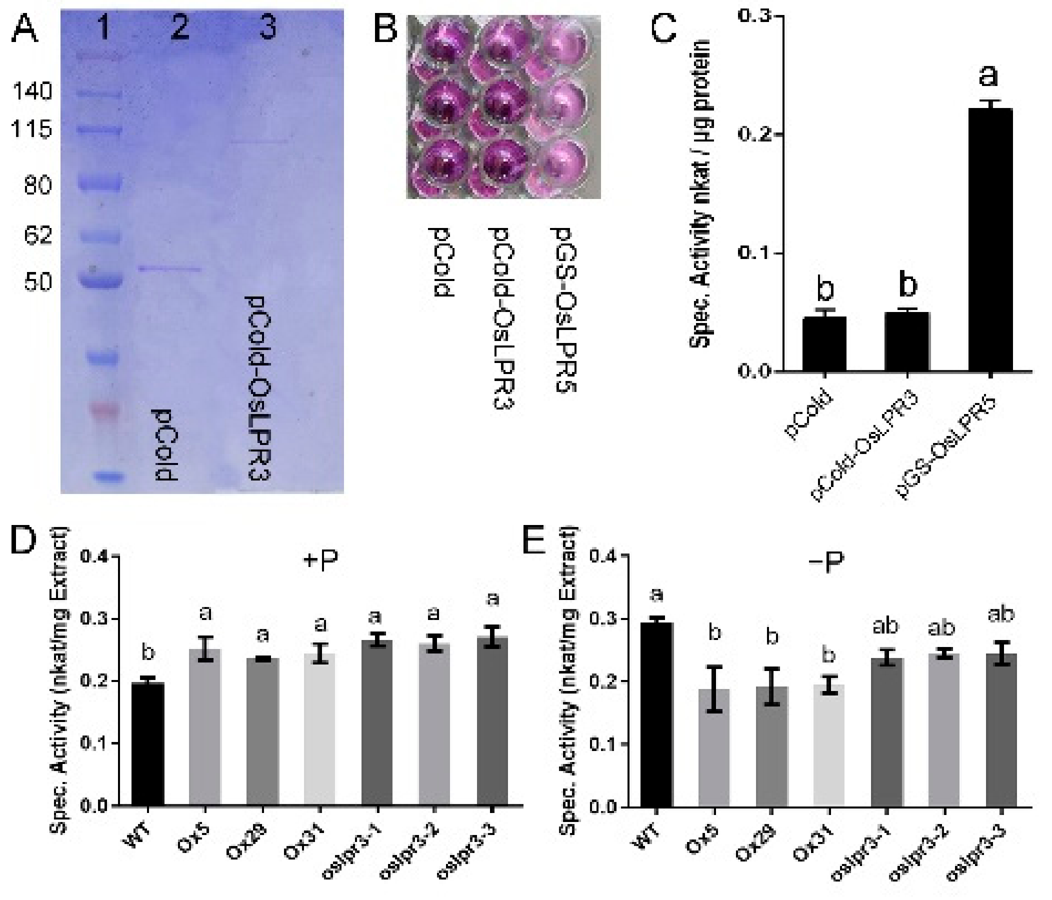

Fig. S8. Ferroxidase activity analysis of OsLPR3.

(A) Purified pCold-OsLPR3 fusion protein was expressed in *E. coli* strain BL21 (DE3) using the vector induced by IPTG and assayed by SDS-PAGE. Purified *pGS*-OsLPR5 fusion protein was used as a positive control. (B and C) Ferroxidase activity was assayed in the BL21 (DE3) strain transformed with pCold (negative control), pCold-OsLPR3, and *pGS*-OsLPR5 (positive control). (D and E) Ferroxidase activity was assayed in the roots of WT and *OsLPR3* overexpression lines (Ox5, Ox29, and Ox31) and *OsLPR3* knockout lines (*oslpr3*-1, *oslpr3*-2, and *oslpr3*-3) grown hydroponically under +P (D) and −P (E) conditions for 2 weeks.

Table S1. The primers used for overexpression, CRISPR, and qRT-PCR

**Gene**		**Sequences**
***OsActin*-qRT- PCR**	F(5'-3')	CAACACCCCTGCTATGTACG
	R(5'-3')	CATCACCAGAGTCCAACACAA
***OsLPR3*-qRT- PCR**	F(5'-3')	TGCATGGTTGTCTCCATTCG
	R(5'-3')	CAAGGCAAACGTACAACAAAGG
***OsIPS1*-qRT- PCR**	F(5'-3')	TTGGCAATTATTCGGTGGAT
	R(5'-3')	ACCATTTCACCATCCTCTTTATG
***OsPAP10*-qRT- PCR**	F(5'-3')	ATACTGGCAGCCGACGGATGA
	R(5'-3')	GAGGGAGCTGGAGCGGAGAA
***OsSQD2*-qRT- PCR**	F(5'-3')	CTGAAAACGGTAATGGATAGG
	R(5'-3')	AACAACAACAGCACGAGC
***OsPT1*-qRT- PCR**	F(5'-3')	CGCTTCCGTACGAGTGGTAGT
	R(5'-3')	GGTTCTTTCAAATCCAGGGAAA
***OsPT2*-qRT- PCR**	F(5'-3')	GACGAGACCGCCCAAGAAG
	R(5'-3')	TTTTCAGTCACTCACGTCGAGAC
***OsPT4*-qRT- PCR**	F(5'-3')	TTCTGCTAGTGTACCAAACAAAATTACA
	R(5'-3')	GTAAGTGGCATTTATAATATCAACAGTAACC
***OsPT6*-qRT- PCR**	F(5'-3')	CCGCCCCTGCAAACTGTA
	R(5'-3')	GAACTGGCGGTTTCTTCGAT
***OsPT8*-qRT- PCR**	F(5'-3')	AGAAGGCAAAAGAAATGTGTGTTAAAT
	R(5'-3')	AAAATGTATTCGTGCCAAATTGCT
***OsPT9*-qRT- PCR**	F(5'-3')	AGAAAAACATAGGCTTGTCATCCTTT
	R(5'-3')	AAAACCTAAGAAGCACTGTAAATAAATCC
***OsPT10*-qRT- PCR**	F(5'-3')	ATGTCGCCCATCCTTCCA
	R(5'-3')	TCGCTTTCCGACGATGATC
35s::OsLPR3	F(5′-3′)	AACGATAGGAGCTCGGTACCTCATGGTCAAGCCTCGTTT
	R(5'-3')	AGGTCGACTCTAGAGGATCCGGGATCACAAGGCAAACGT
OsLPR3-CRISPR-S1	F(5′-3′)	GGCATTCTCCCACGTCACGTAGAG
	R(5'-3')	AAACCTCTACGTGACGTGGGAGAA
OsLPR3-CRISPR-S2	F(5′-3′)	GGCAGCCCAGGCCCCCGATTCCGA
	R(5'-3')	AAACTCGGAATCGGGGGCCTGGGC
